# Diet-Microbe-Host Interactions That Affect Gut Mucosal Integrity and Infection Resistance

**DOI:** 10.3389/fimmu.2019.01802

**Published:** 2019-08-06

**Authors:** Andrew J. Forgie, Janelle M. Fouhse, Benjamin P. Willing

**Affiliations:** Department of Agricultural, Food and Nutritional Science, University of Alberta, Edmonton, AB, Canada

**Keywords:** microbiota, diet, infection resistance, gastrointestinal integrity, disease susceptibility

## Abstract

The gastrointestinal tract microbiome plays a critical role in regulating host innate and adaptive immune responses against pathogenic bacteria. Disease associated dysbiosis and environmental induced insults, such as antibiotic treatments can lead to increased susceptibility to infection, particularly in a hospital setting. Dietary intervention is the greatest tool available to modify the microbiome and support pathogen resistance. Some dietary components can maintain a healthy disease resistant microbiome, whereas others can contribute to an imbalanced microbial population, impairing intestinal barrier function and immunity. Characterizing the effects of dietary components through the host-microbe axis as it relates to gastrointestinal health is vital to provide evidence-based dietary interventions to mitigate infections. This review will cover the effect of dietary components (carbohydrates, fiber, proteins, fats, polyphenolic compounds, vitamins, and minerals) on intestinal integrity and highlight their ability to modulate host-microbe interactions as to improve pathogen resistance.

## Introduction

Infectious enteric diseases are a major cause of morbidity and mortality worldwide and are of particular concern in hospital settings and developing countries. According to the World Health Organization, infectious enteric diseases are one of the top 10 causes of death leading to over two billion cases and one million deaths worldwide in 2010 (1). Host resistance toward invading pathogens requires tight regulation of the gastrointestinal environment, maintained through a synergistic relationship between the host immune system and microbiome. Disruption to a host's intestinal homeostasis, including insults from diet, stress, antibiotic and drug treatment, allergies, cancer, and related illnesses can leave the host vulnerable to enteric pathogens (2). It is well-understood that diet can play a major role on health by positively and negatively shaping gastrointestinal ecology (3, 4), and therefore should be a major focus in mitigating the severity of infection.

Although humans have successfully reduced pathogen exposure through effective sanitation practices, the adoption of a “Western diet,” over-sanitation and lack of physical exercise are hypothesized to have contributed to the rise in autoimmune disorders (5). The “Western diet” is characterized by the excessive consumption of fats, proteins, refined sugar, and low intake of dietary fiber. Other dietary patterns such as the Mediterranean, Vegetarian-based, Japanese-based, and Ketogenic type diets can positively regulate immune responsiveness to reduce immune activity and support health (6). However, human epidemiology studies on diet tend to exclude important interindividual variations that govern the gastrointestinal microbiota and may explain the diverse claims to which foods are known as “protective” and “harmful” (7). Establishing a mechanistic link between individual diet components using microbe-host interactions will aid to provide evidence driven recommendations to help control an overactive immune response.

An overactive immune system is associated with autoimmune disorders such as irritable bowel disease (IBD) that affects host immune activity and leads to increased incidence of infection (8, 9). Likewise, “westernized diets” have shown to enhance *Escherichia coli* colonization and associated inflammation in mice by altering the host mucus layer, increasing intestinal permeability, and impairing immune function (10). Dietary fiber and other microbiota-accessible carbohydrates (MACs) are a key component missing from the “westernized diet” that when re-introduced provides a beneficial balance to host health and microbiome (11). Fiber is exhaustively studied as a microbial fermentation substrate that produces short chain fatty acids (SCFAs) with known benefits to host intestinal homeostasis and health (12). However, we fear that this focus on the beneficial effects of fiber-associated SCFA production has led researchers to overlook other common dietary components that may positively or negatively influence the host gastrointestinal environment and health.

**Figure 1:**
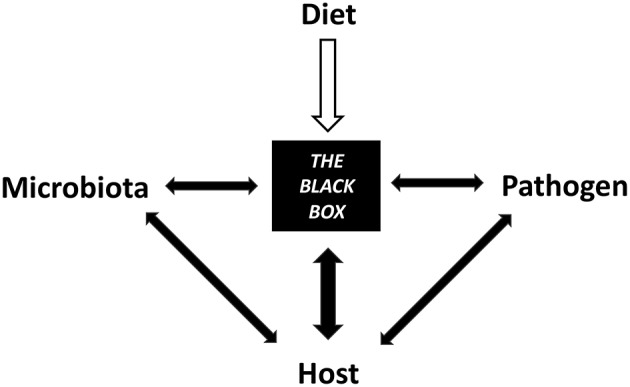
Diet contributes to a black box of intertwined mechanisms between the microbiota, host, and pathogen that have yet to be elucidated.

Diet intervention should be considered a valuable tool to manipulate the host-microbe axis to help sustain intestinal homeostasis and infection resistance. Dietary components such as carbohydrates, lipids, proteins, phytochemicals, minerals, and vitamins all have unique structural and chemical (physicochemical) properties that influence host pathogen resistance directly and indirectly through the microbiome. Bridging the gap between diet, host, and microbiome as they relate to immunity and disease resistance is a multifaceted field that requires an understanding of their combined effects on intestinal homeostasis (Figure 1). This review explores the role of common dietary components on host-microbe interactions that modulate host resistance and tolerance toward common infectious diseases. We highlight the opportunity to improve outcomes, yet recognize the current knowledge limits the ability to provide concrete dietary advice. This is partially limited by the fact that diet focused infection resistance research is scarce and difficult to translate to humans.

## GALT and Microbiome Regulate Host Defenses

The gut associated lymphoid tissue (GALT) plays a crucial role in regulating intestinal homeostasis and is composed of lymph nodes, lamina propria, and epithelial cells that together provide the host with a protective barrier and immune defense against invading pathogens (13). On the other hand, the microbiota provides a physical presence that can directly prevent pathogen colonization by competing for attachment sites or nutrient resources. Indirectly, the microbiota helps to improve host resistance by modulating intestinal integrity through the mucus layer, tight junction proteins, and antimicrobial peptides (AMPs: cathelicidins, C-type lectins, and defensins) (14, 15). Mucins secreted by goblet cells provide the first line of defense by forming a physical barrier composed of highly glycosylated and interlinked proteins between luminal bacteria and host epithelial cells (16). The mucus layer provides lubricant and is metabolized by mucin-degrading (mucolytic) bacteria forming the loosely attached layer (17), whereas the adherent layer, when properly formed, secures a balance of host AMPs and immune factors that maintain intestinal homeostasis (18).

Disruptions to the balanced microbial ecosystem greatly increase a host's vulnerability to infection (19). In particular, antibiotic exposure can cause major shifts in microbial communities leading to mucus layer thinning, predisposing, and exacerbating infections, as shown with antibiotic accompanied *Citrobacter rodentium* infections in mice (20). Antibiotic-induced microbiota imbalances are well-documented to alter the production of AMPs, tight junction proteins, and immune factors that normally contribute to intestinal homeostasis and infection resistance (21, 22). Secretory immunoglobulin A (SIgA) antibodies are abundant immune factors of the intestinal lumen that protect epithelial cells from enteric pathogens and toxins by blocking their access to epithelial receptors and entrapping them in mucus to promote clearance (23). Although SIgA targets and disrupts pathogens and antigens, commensal microbes such as *Bacteroides fragilis* alter their surface proteins to attract SIgA to enhance mucosal colonization (24). Intestinal epithelial cells (IECs) produce reactive oxygen species (ROS) (25) and Resistin-like molecules (e.g., RELMβ) (26) that hinder commensal and pathogenic bacteria colonization, further maintaining intestinal hemostasis. IECs apical surface fucosylation is another useful host strategy that controls commensal microbes and inhibits pathogens. Secreted fucose is metabolized by bacteria to produce bioactive metabolites, reduce virulence factors, and enrich beneficial gut microbes to strengthen colonization exclusion (27). Alternatively, fucose can be fermented by commensal microbes into 1,2-propanediol and utilized by *Salmonella* during inflammation to drive their fitness in the colon (28).

**Figure 2:**
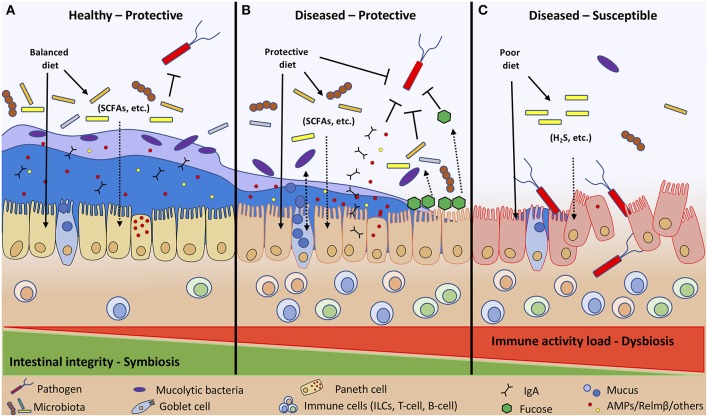
Diet and immune activity load (allergies, cancer, other illness, etc.) determine host intestinal integrity toward invading pathogens. Diet affects intestinal integrity directly by stimulating IECs, ILCs, and microbial communities, and indirectly through microbial fermentation by-products (SCFAs, H_2_S, etc.). A healthy individual following a balanced diet to maintain symbiosis between host and microbial populations has enhanced intestinal integrity with a thick inner and outer mucus layer that retains AMPs and other compounds to protect the host against pathogen colonization **(A)**. A diseased host with heightened immune activity maintains symbiosis by consuming dietary components that protect and boost host innate defenses (IgA, AMPs, mucus, fucosylation) and adaptive immune responses to prevent pathogen colonization **(B)**. Whereas, diseased individuals with heightened immune activity consuming a poor diet are more susceptible to enteric infections due to impaired host defenses that cannot control the dysbiotic intestinal environment **(C)**.

The host has significant control over microbial communities of the small and large intestine; however, this relationship is complex and is managed in part through gastric acid secretions, intestinal motility, bile secretions, oxygen gradients, and regulation of pattern recognition receptors (PPRs), such as Toll-like receptors (TLRs) (4). The host recognizes commensal bacteria through activation of TLRs and relays an appropriate response in accordance to the specific microbial derived ligands [e.g., peptidoglycan, lipoprotein, lipopolysaccharide (LPS), and flagellin] (29). Innate lymphoid cells (ILCs) have been identified as key immune regulatory cells of the GALT controlling pathogen resistance, inflammation, and metabolic homeostasis (30). ILCs concentrate within mucosal surfaces and relay signals sent between the microbiota, epithelia, immune cells, and metabolites in the intestine to maintain epithelial barrier function. Transcriptomic analysis of 15 ILC subtypes revealed their regulatory functions depend on the presence of the microbiome, nutrients, and xenobiotics (31). Ultimately, it is the combined relationship between the gut microbiota, host, and diet that help improve or worsen a host's ability to tolerate and resist pathogenic bacteria (Figure 2). The remainder of this review will focus on specific dietary components and how they stimulate some of these and other host-microbe interactions resulting in impaired or improved host disease resistance.

## Carbohydrates

Dietary carbohydrates are often classified by their degree of polymerization into mono-, di-, oligo-, or poly-saccharides and composition of their monosaccharides: glucose, fructose, galactose, and xylose. Typically, carbohydrates are categorized as either digestible or indigestible (fiber). Binding and structural properties of carbohydrates dictate the glucosidase enzymes required to break bonds into their basic units for absorption (32). The digestible carbohydrates escaping host small intestinal digestion, along with dietary fiber, become available as microbial energy substrates and are able to substantially alter the intestinal ecosystem and community structure (33).

Increasing intake of digestible carbohydrates has been scrutinized for contributing to the worldwide obesity and diabetes epidemics. However, carbohydrates are essential energy substrates for the central nervous system and red blood cells, are required to maintain cellular energy balance after sustained increases in metabolic activity, and to restore energy levels and glycogen stores (34). Humans and animals are able to regulate blood glucose levels; however, excessive dietary carbohydrate consumption can worsen acute hyperglycemia, particularly during times of an illness (35, 36) and stress (37, 38). A medical illness can enhance the negative effects of acute hyperglycemia, which include inhibition of neutrophil migration, phagocytosis, superoxide production, and microbial killing, compromising host innate immunity against bacterial and fungal infections (39). Diets high in simple and refined carbohydrates are shown to negatively impact gastrointestinal microbial communities leading to intestinal barrier dysfunction and greater risk for enteric infection (36). Whereas, balanced diets containing resistant starch and fiber stimulate microbial fermentation leading to a stable diverse microbiome and production of beneficial SCFAs (40). Understanding both negative and positive effects of carbohydrate consumption on gastrointestinal immunity and microbial populations will provide vital insight toward dietary strategies to help maintain pathogen resistance.

Dietary trehalose, a food component used to improve a product's texture, flavor, glycemic index and shelf life, was introduced in the early 2000's and has since been proposed to have contributed to the global *Clostridioides difficile* epidemic (41). Trehalose is a disaccharide composed of two glucose molecules linked by a resistant α,α−1,1-glucosidic bond found in plants, algae, fungi, yeast, bacteria, insects, and other invertebrates (42). Mammals and other vertebrates lack the ability to synthesize trehalose, and the dietary fate of trehalose depends on the capacity of the small intestinal trehalase enzyme to hydrolyze it into glucose (43). Trehalase deficiency is rare in humans but excessive consumption of trehalose can lead to negative intestinal imbalances similar to those associated with lactose and fructose intolerances. Researchers believe the increased use of trehalose in food production has naturally selected for *C. difficile* with the capacity to metabolize trehalose more efficiently, thus increasing pathogen fitness and contributing to their hypervirulent outbreaks in the human population (41). To combat reoccurring *C. difficile* infections a fecal microbial transplant (FMT) from a healthy donor has become a helpful treatment option, however the mechanism of remission remains unclear (44). The success of FMTs to treat *C. difficile* infections highlights the importance of a “healthy” gut microbiome to promote infection resistance. Additional research is needed to confirm the impact of specific carbohydrates and their malabsorption on immune and microbial networks in the gut as it relates to pathogen fitness. Interestingly, studies in mice comparing fiber-rich and fiber-deprived diets support the detrimental effect of a simple carbohydrate dominated diet and the importance of fiber on infection resistance (11, 33).

## Dietary Fiber

Health benefits associated with foods rich in non-digestible dietary fiber depend on their type, source, and proportion of water soluble and insoluble carbohydrate components (45). Fruits, vegetables, and grains are excellent sources of numerous fiber types, however, not all fiber sources and types are created equal. The food source, glycosylated chain structures, and their fermentability, along with other inherent components are key parameters for their functional quality within the gastrointestinal tract (12). Non-digestible carbohydrates are composed of monosaccharide units (glucose, fructose, galactose, xylose, fucose, and sialic acid) found naturally in plants, algae, fungi, bacteria, and mammalian milk, or produced by chemical or enzymatic processes (46, 47). Short chain fructo-oligosaccharides (FOS) have received a great deal of attention due to their prebiotic effects (48) and fact that they occur naturally (mostly as inulin) with different degrees of polymerization in foods (47). The consumption of prebiotic fibers have helped with diarrhea and constipation (49–51), however, not everyone benefits from their consumption, and can even lead to excessive gas production, bloating, and discomfort (50, 52). In cases of gastrointestinal discomfort, a diet low in fructans (FODMAP-restricted diet) or reducing dietary fiber is often effective but remains controversial, and individualized (53–55).

The effects of various non-digestible fiber on health and microbiota is thoroughly reviewed (12, 45, 46). In general, dietary fiber can modify gastrointestinal function directly through fecal bulking and indirectly through the modification of microbial community structure, and by increasing microbial biomass and fermentation products (45). Fiber fermentation leads to beneficial SCFAs (mainly acetate, propionate, and butyrate) but also undesired gases such as carbon dioxide, hydrogen, and methane (56). Increased gas production, fecal bulking and delayed gastric emptying can lead to discomfort, bloating, and flatus in many individuals (45). Microbial fermentation products such as SCFAs interact with the intestinal epithelium to promote certain defense mechanisms. In particular, microbial production of butyrate provides an energy substrate to epithelial cells (57), maintains the hypoxic environment (58), and promotes improved barrier function through hypoxia inducible factor (HIF) (59). Induction of HIF transcription factor subsequently stimulates downstream signaling to increase mucus production (60) and expression of AMPs (61) ultimately helping to minimize facultative pathogen growth.

According to the Global Burden of Diseases, Injuries, and Risk Factors Study of 2015 infectious diarrhea is a leading cause of death globally among all ages (1.3 million deaths); with a large proportion of those occurring in infants under 5 years of age (499,000 deaths) (62). Providing children with MACs is an important strategy to mitigate infection burden by stabilizing the microbiota and by bolstering intestinal immunity. Infants that are exclusively breast fed have reduced risk of developing diarrheal disease (63), partially due to the naturally occurring human milk oligosaccharides (HMOs) present in breast milk. HMOs are soluble complex carbohydrates that act as prebiotics, providing a substrate for the intestinal microbiota and can prevent pathogenic bacterial adhesion through a variety of mechanisms (64). *In vitro* studies determined that HMOs act as pathogen decoy receptors to prevent infections and their activities depend on the location and degree of fucosylation (65). Human breast milk contains a multitude of other bioactive factors, immunoglobulins, cytokines, chemokines, growth factors, hormones, and lactoferrin which all likely contribute to the improved disease resistance of breast fed infants and is reviewed elsewhere (66). Human milk has shown the ability to directly inhibit the adherence of *Streptococcus pneumonia* and *Haemophilus influenza* to human mucosal cells *ex vivo* (67). When HMOs were fractioned, it was found that the acidic fraction had greater anti-adhesive properties toward enteropathogenic *E. coli* (EPEC)*, Vibrio cholera*, and *Salmonella fyris* compared to the neutral high and low molecular weight fractions (68). Similarly, HMOs blocked EPEC adherence to epithelial cells *in vitro* and reduced EPEC colonization in newborn mice, further implying the essential role HMO play in the prevention of infectious disease in human infants (69). Experimentally, it was shown that supplementing formula with HMO reduced the duration of diarrhea in rotavirus-infected pigs and promoted IFNγ and IL-10 expression in the ileum, suggesting HMOs may also protect infants against rotavirus infection (70). Therefore, research efforts have focused on HMO substitutes that can be added to formula fed to infants that are unable to breastfeed. Human and animal studies suggest supplementing formula with fermentable fiber (e.g., soy polysaccharides, fructo- & galacto- oligosaccharide) reduces infection-associated diarrhea burden by improving intestinal homeostasis (71) and increasing beneficial *Bifidobacterium* species (72–74).

Minimizing infectious diarrheal disease with dietary tools has become the focus of recent research efforts. The importance of non-digestible fermentable fiber or MACs intake in adults has clearly been shown where a greater intake (comparing top vs. bottom quartiles) reduced risk of death from cardiovascular, infectious, and respiratory disease by 24–56% in men and 34–59% in women (75). Galacto-oligosaccharides (GOS) have shown to increase bifidobacteria and beneficially modulate immune function when supplemented to elderly volunteers. Along with improving phagocytosis and natural killer cell activity, the GOS supplemented volunteers had an anti-inflammatory cytokine profiles with increased IL-10 and reduced IL-1B, IL-6, and TNFα (76). In a double blind placebo controlled trial, those supplemented with GOS had reduced diarrhea incidence, duration, and severity (77). *Clostridioides difficile* is the leading cause of health care-associated diarrheal infections, commonly affecting the elderly and antibiotic treated hospitalized patients (78). Significant evidence suggests that the inclusion of soluble fiber to the diet, specifically MACs that increase SCFA production, may be a useful strategy to enhance infection resistance (79). In a mouse model, dietary inclusion of MACs or inulin alone was shown to suppress *C. difficile* infection; whereas diets devoid of MACs exacerbated the infection (11). The mechanisms by which MACs help to mitigate *C. difficile* infection is through the expansion of fiber fermenting microbiota (via competitive exclusion) and subsequent increases in their immune-stimulatory metabolites (promote host defenses), which limit a pathogen's fitness (11).

β-glucans are one type of fermentable fiber that is frequently studied due to its common occurrence in the cell walls of yeast, fungi, and cereals such as barley and oats. Aside from acting as a microbial fermentation substrate, β-glucans are also of great interest for their direct effect on host immune activities and functions that alter immunity toward infections. In humans, the immune modulating property is due to the binding of β-glucans with host receptor dectin-1 (80), which contributes to macrophages activation, and induce phagocytosis (81). Studies in mice found that oat derived β-glucans supplemented at 3 mg every other day stimulated a systemic immune response that reduced fecal oocyst shedding of *Eimeria vermiformis* by 39.6% post-challenge by increasing specific antibodies against the parasite (81). Oral administration of β-glucan from a fungal source (*Sclerotinia sclerotiorum* at 80 mg/kg every 2 or 3 days) was shown to directly stimulate proliferative responses of Peyer's patches to both T and B-cell mitogens, suggesting β-glucans may also stimulate a mucosal immune activation (82). Intraperitoneal injection of β-glucans has also shown to work as a potent adjuvant to enhance host resistance to both bacterial (81) and parasitic (Leishmania) infections (83). The use of immunostimulants derived from naturally occurring polysaccharides (e.g., β-glucan or chitosan) has become somewhat commonplace in the aquaculture industry as an alternative strategy for disease prevention. Inclusion of oligo-β-glucans (100–200 mg/kg) to striped catfish has shown to improve growth performance and reduce mortality post *Edwardsiella ictaluri* challenge via heightened phagocytic and lysozyme activity (84). The inclusion of dietary β-glucans (200 mg/kg) in poultry has also been used effectively to reduce the severity of necrotic enteritis when challenged with *Eimeria* and *C. perfringens* (85) and inhibited growth depression when challenged with *Salmonella enteritidis* (86) by increasing specific antibody levels. In both cases, inclusion of dietary β-glucans reduced pathogen colonization (*C. perfringens* and *S. enteritidis*).

Generally, increasing fiber will change the microbiome and improve gastrointestinal heath. As stated previously, the benefits associated from consuming food sources or supplements high in fiber is individualized and should be carefully monitored for side-effects.

## Fats

Fats are an essential dietary macronutrient that have been criticized and are commonly avoided in developed countries with the objective of reducing weight, cholesterol levels, and cardiovascular disease risk. Fat avoidance and subsequent reliance on simple carbohydrates for caloric intake with reduced energy expenditure is believed to have contributed to the unintended rise of obesity worldwide (87). In healthy individuals most fats are emulsified and absorbed in the small intestine; however, in excess and during intestinal stress fats can travel toward the colon as a substrate for the microbiota (88). Human and animal studies have shown that intestinal microbes have the capacity to alter host homeostasis through a variety of metabolites, including carcinogenic and cytotoxic secondary bile acids (89). The effects of the microbiota on host homeostasis is through alteration to hepatic lipid and bile metabolism, reverse cholesterol transport, energy expenditure, and insulin sensitivity in peripheral tissue (90). In this respect, dietary lipids are capable of directly affecting the host and microbiome, while indirectly altering host homeostasis through the microbiome and their metabolites.

The direct effect of microbial fat metabolism on intestinal health has yet to be established but studies have shown that dietary lipid profiles can alter the outcome of enteric infections. Fat consumption with regards to infection have been thoroughly reviewed elsewhere (91), and provides a bases to establish the connection between microbe and host on enteric pathogen resistance. A study comparing dietary saturated (SFA, milk), monounsaturated (MUFA, olive oil), and polyunsaturated (PUFA, omega-6 corn oil) fatty acids uncovered distinct lipid mediated immune responses in mice after an acute *C. rodentium* challenge (92). SFA and MUFA dominated diets induced protective T-regulatory cells, interleukin (IL)-10, IL-33, and SCFAs that helped mitigate inflammation during enteric infection (92). Interestingly, in a dextran sodium sulfate (DSS) model, IL-10 knockout mice fed a diet containing milk SFAs, but not lard fat SFAs, resulted in a pro-inflammatory T_H_1 immune response associated with a bloom of *Bilophila wadsworthia* and its metabolites, hydrogen sulfide and secondary bile acids (93). Diets high in medium-chain SFAs like coconut oil have antifungal action toward *Candida albicans* (94) and antibacterial properties against enteric pathogens (95). Moreover, the addition of fish oil, high in omega-3 (n-3) fatty acids to a SFA dominated diet activated intestinal alkaline phosphatase (IAP), an enzyme that detoxifies proinflammatory lipopolysaccharide (LPS) endotoxins from gram-negative bacteria that accumulates during infection; whereas supplementing n-3 to an n-6 rich diet did not enhance IAP activity (92). Previously it has been observed that high levels of dietary n-6 PUFAs in fact reduce IAP activity leading to LPS endotoxemia in mice (96). Transgenic *Fat-1* mice, which genetically retain a higher concentration of n-3 in their tissues, demonstrated elevated serum IL-10 and IAP activity (96). In mice, safflower and canola oil based diets (high in n-6) heighten mucosal T_H_1/T_H_17 responses and inflammation, whereas a fish oil based diet has shown to have a protective anti-inflammatory effect following a *C. rodentium* infection (97). Diets rich in n-3 PUFAs have proven protective against many extracellular pathogens (*Mycobacterium tuberculosis, Salmonella typhimurium, S. pneumoniae, Pseudomonas aeruginosa, E. coli, Staphylococcus aureus, C. rodentium, Helicobacter hepaticus, H. pylori*, and *Listeria monocytogene*); however, potentially damaging effects were observed during intracellular viral infections (98, 99). Dose and timing of n-3 PUFAs is critical for intestinal immune homeostasis. Sustained high doses alter microbial communities and host immune system toward an anti-inflammatory state that could exacerbate infections, especially when proinflammatory responses are essential for infection clearance (98). Interestingly, lipid composition affects host-microbial interactions even when administered via a non-enteral route. The inclusion of mixed lipids containing soybean oil, medium-chain triglycerides, olive oil, and fish oil in parenteral formula was shown to reduce intestinal inflammation and alter microbial composition in a piglet model of infant total parenteral nutrition as compared to soybean oil alone (100).

## Protein

Protein homeostasis is crucial for host health, physiology, and immune development that together foster a fast-acting immune response toward pathogens. The role of dietary protein and amino acids on host immune function related to diet malnutrition and pathogen interactions has been thoroughly reviewed (101, 102). Amino acids play a major role in regulating immune cell activation, cellular redox homeostasis, lymphocyte proliferation, and production of cytokines, cytotoxins, and antibodies (101). Protein deficiency is well-known to impair immunity and infection resistance, especially during stress and illness due to protein malabsorption and protein consuming processes such as tissue repair (103). Protein deficits have been shown to exacerbate parasitic *Cryptosporidium* infections in mice through disruption of baseline (primary) Th1-type mucosal immunity (104). Furthermore, protein-deprived diets decreased small intestinal macrophage proliferation and IL-10 production independently of the microbiota (105).

In contrast, researchers propose that protein-rich diets can be just as harmful since they lead to an increase in undigested proteins that encourage protein-fermenting bacteria and disease susceptibility (106). Resistant and undigested proteins can interfere with host functions directly as biologically active proteins (BAP) like trypsin and chymotrypsin inhibitors, and indirectly through microbial proteolytic fermentation by-products [H_2_, CO_2_, CH_4_, H_2_S, SCFA, branched chain amino acids (BCAA), nitrogenous compounds, phenols, and indoles] with poorly understood health outcomes (107). It is important to note that dietary crude protein can contain a high concentration of BAPs whose activities can be reduced upon hydrolysis digestion (heating, chemical, or enzymatic). A study replacing crude protein (wheat and casein) with purified amino acids to diets fed to weaned pigs reduced proteolytic fermentation before and after an enterotoxigenic *E. coli* (ETEC) K88 challenge (108). Three days post-infection, ETEC K88 colonized the small intestine of pigs fed the crude protein diet whereas no colonization was observed in the small intestine of pigs receiving the purified amino acid diet. In this context, undigested protein or other components associated with crude protein diets promoted ETEC growth and colonization in the small intestine.

Furthermore, the source of proteins can impact microbial communities depending on the digestibility and total amino acids in the diet (106). For instance, animal proteins tend to be highly digestible in the proximal intestine compared to plant-based proteins (109). Processing proteins with heat can impact their digestibility, for example, rats fed thermolyzed (heated to 180°C for 1–2 h) casein, soy, or egg white protein had reduced proximal intestinal digestibility, leading to a greater degree of protein fermentation in the cecum (110). The number of aberrant crypts were measured after azoxymethane challenge to assess the carcinogenic promoting properties of casein, soy, and egg proteins. For the heat-treated proteins, the number of aberrant crypts increased with casein, remained unchanged with soy, and decreased with egg white compared to untreated protein diets. In agreement, a DSS mouse model study using multiple custom diets demonstrated that casein and soy proteins worsened DSS associated weight loss, whereas no effect was seen in mice fed the egg white protein diets (111). In contrast, a human trial compared high- and low-fat diets with non-meat protein (legumes, nuts, grains, soy), red meat protein (beef) or white meat protein (chicken and turkey) on the gut microbiome and found only a modest impact of protein source on the microbiome (112). For cardiovascular health, the plant-based proteins outperformed meat protein diets but white meat was no better than red meat for reducing disease risk (113). However, animal protein dominated diets tend to include higher amounts of fats, which ultimately may be more impactful on health than the proteins themselves. Plant-based protein diets may inherently contain detrimental components. For example, soybean isoflavones are suggested to contribute to greater parasitic oocyst fecal output and reduce immune responsiveness in mice fed a soy-based diet compared to casein and whey protein fed groups (114). For this reason, crude protein diet studies make it difficult to identify the bioactive component responsible for the observed phenotype. A study in rats comparing protein from soy, casein, pork, beef, chicken, and fish indicates that protein source alters microbial composition (115). Specifically, white meat (chicken and fish) increased beneficial *Lactobacillus* species. Blood levels of lipopolysaccharide-binding protein (LBP), a marker for lipopolysaccharide (LPS) endotoxemia, was found to be significantly higher in the soy protein diet group compared to fish, chicken, pork, beef, and casein protein fed groups. Further research is needed in controlled animal models to investigate isolated protein types and processing techniques on host digestion, microbiome, and fermentation products to mechanistically link the impact of protein on infection resistance.

Dietary glutamine supplementation has proven to be an effective therapy to help restore intestinal integrity in patients with post-infectious associated irritable bowel syndrome (116). Although glutamine significantly improved IBS scores compared to a placebo supplemented group, a larger cohort and mechanistic studies are warranted. The effect of glutamine supplementation may be associated with glutamines ability to enhance intestinal cell proliferation (117), decrease the Firmicute population, and activate innate immunity through NF-κB, MAPK, and PI3K-Akt signaling pathways (118). Similar effects have been observed with arginine supplementation (119). Over a 14-day study, daily supplementation of 30 g of L-glutamine to overweight individuals led to a significant decrease in Firmicute populations, including species from the genus *Dialister, Dorea, Pseudobutyrivibrio*, and *Veillonella* (120). Since overweight individuals typically have a higher Firmicute/Bacteroidetes ratio than lean individuals (121), a decrease in Firmicutes with glutamine supplementation suggests that dietary glutamine may play a beneficial role in restoring microbiota balance. In accordance, glutamine and arginine supplementation promoted the activation of innate immunity and lowered intestinal pathogen load in ETEC-infected mice (122). In humans, enteral glutamine administration in critically ill patients with severe trauma, burns, and sepsis significantly reduced the number of isolated enteric bacteria such as *Pseudomonas* sp., *Klebsiella* sp., *E. coli*, and *Acinetobacter* sp., all of which can contribute to pneumonia if transmitted to the lungs (123, 124). Enteral glutamine administration reduced bacterial overgrowth within the gastrointestinal tract, which may have reduced the chance of bacterial exposure to the lungs and explain the reduced incidence of pneumonia in patients. Moreover, a systematic review and meta-analysis concluded that glutamine-enriched enteral formulae can significantly reduce gut permeability in critically ill patients (125). The requirement and importance of enteric glutamine has been extensively reviewed (126), but requires further research in healthy subjects and animals models to understand the impact on the microbiome and enteric infection resistance.

**Figure 3:**
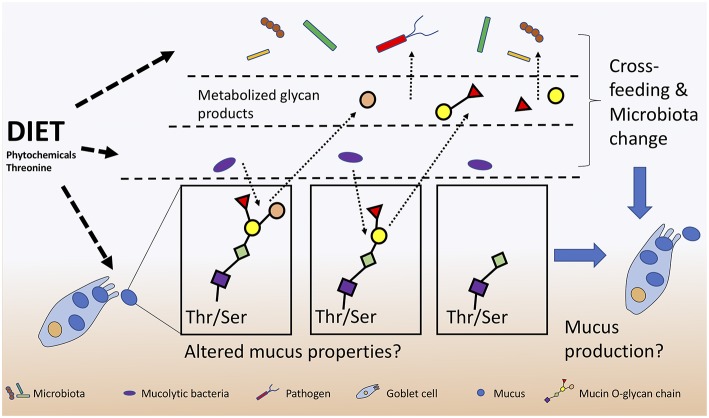
Diet alters host-microbiota-pathogen mechanisms of mucus production and consumption. Mucolytic specialists that digest the mucus O-glycans and subsequently cross-feed with other bacteria and pathogens can lead to further microbiota changes and alterations to mucosal integrity.

Further emerging evidence suggests that numerous microbially-derived indoles from tryptophan catabolism can promote intestinal homeostasis by activating regulatory T cells (Tregs) through their interaction with the aryl hydrocarbon receptor (AhR) (127). Roager and Licht summarize known microbes responsible for producing tryptophan-derivatives that positively act on tight junctions, gastrointestinal motility, host metabolism, AhR to activate IL-22, along with their systemic anti-oxidative and anti-inflammatory properties (128). In this respect, dietary tryptophan likely contributes to infection resistance by priming host defense strategies. The importance of tryptophan is further supported by the ability of host dendritic cells to metabolize tryptophan into kynurenine using indoleamine 2,3-dioxygenase-1 (IDO1) in order to control host inflammation during a *C. difficile* infection (129). Kynurenine production during *C. difficile* infection is proposed to be beneficial as it reduces excessive interferon-γ (IFNγ) cytokine production by limiting neutrophil populations in the lamina propria (129). Clinically, these findings provide important insight into the use of IDO1 inhibitors for cancer treatment which would prevent kynurenine production, and increase the severity of *C. difficile* infection (129). Like tryptophan, threonine is another essential amino acid that must be obtained from diet with deficiencies leading to immune and barrier dysfunctions (130). Dietary threonine is essential for the production of mucin with deficient diets leading to altered mucosal integrity and persistent diarrhea in neonatal piglets (131). The importance of dietary threonine for mucus production and structure may not only provide protection for host IECs but also could stimulate mucolytic bacteria with unknown functions (Figure 3).

Dietary protein source, amount, and processing can alter their impact and effects within gastrointestinal environment. Clearly host protein digestion shares an intimate relationship with the gut microbiome and their fermentation products (132). A balanced macronutrient or low indigestible protein diet is recommended to discourage proteolytic bacteria from overproducing cytotoxic, genotoxic, and carcinogenic by-products that disrupt intestinal integrity and increase the risk of infection (106).

## Phytochemicals

Plants synthesize a large pool of compounds known as phytochemicals to protect themselves from stress, predation, and infection. Complex mixtures of phytochemicals are found in the roots, seeds, leaves, bark, flowers, and fruit of plants and have been intensively studied for their antimicrobial, anti-inflammatory, and antioxidants activities (133). The physicochemical properties of phytochemicals give plants their unique color, smell, and flavor profiles, and dictates their bioactivities and bioavailability within the gastrointestinal tract (134). Condensed tannins, mainly polymeric flavanols can act as antinutritional factors that reduce host digestion through enzyme inhibition and protein precipitation (135). However, the consumption of phytochemicals is typically associated with beneficial health outcomes from their activities on the resident microbial population and host metabolism (14, 136, 137). Phytochemicals are treated as xenobiotics by the host and because of this, the liver can reintroduce phytochemical derivatives to microbes through enterohepatic circulation, further complicating their effects on host health. Many studies fail to demonstrate and characterize absorbed phytochemical derivatives to investigate whether their impact on host are direct or indirect through the microbiota.

Research has focused on the use of phytochemicals as an alternative to antibiotics and as a dietary supplement to strengthen host pathogen resistance (138). For instance, chickens fed a mixture of pepper (*Capsicum*) and turmeric oleoresin had less weight loss and reduced intestinal lesions scores in a necrotic enteritis disease model (139). The phytochemical mixture lowered intestinal but increased splenic proinflammatory cytokines/chemokines (IL-8, lipopolysaccharide-induced TNF-a factor, IL-17) levels altering host immunity through immune cell differentiation, proliferation, apoptosis and NO production (139). Reactive nitrogen and oxygen species produced by peripheral leucocytes is an essential defense strategy against pathogens. In fish, dietary supplementation of a grass extract (*Cynodon dactylon*) to infected *Catla catla* carp stimulated reactive oxygen and nitrogen species production and decreased mortality in a dose depend manner (140). Screening multiple phytonutrients revealed that the dietary flavonoid naringenin can act as an agonist on the AhR to induce regulatory T cells (Treg) that suppress allergy and autoimmune disease (141). Interestingly, phytochemicals such as indole-3-carbinol (I3C) present in cruciferous vegetables (e.g., broccoli, cabbage) act as ligands for AhR leading to the expansion of the anti-inflammatory IL-22 producing ILCs (142). Functioning AhR has proven to be crucial for immunity because AhR-deficient mice failed to control *C. rodentium* infections (143). Moreover, mice fed a phytochemical-free diet had a reduced formation of lymphocyte aggregates and follicles, a similar phenotype as seen in AhR-deficient mice (142). Dietary I3C supplementation protected against *C. difficile* infection through activation of AhR but also through unknown AhR-independent mechanisms likely caused by changes to microbial populations (144).

Anti-adhesion properties are well sought after when studying the direct effects of phytochemicals on pathogen fitness. Cranberry extracts are documented to inhibit pathogenic *E. coli* adhesins (e.g., fimbriae) limiting their ability to attach to host cells (145, 146). The anti-adhesion activity of cranberry extract is attributed to the polyphenolic flavan-3-ol compounds known as A-type proanthocyanidins (PACs) (147). Cranberry A-type PACs reduced adherence of multiple strains uropathogenic *E. coli* and *Proteus mirabilis in vitro* (145). However, *in vivo*, intestinal and microbial PACs metabolites are found at higher concentrations in urine than the intact PACs and thus may be the bioactive metabolites responsible for the anti-adhesive properties (148). Interestingly, an analysis of urine phytochemical metabolites indicated that they change over-time due to multiple rounds of enterohepatic circulation modifications (148) with poorly understood activities (149). Moreover, cranberry PACs are thought to inhibit host and microbial enzymes (e.g., lipase, glycosidases) protecting against diet-induced obesity (150). PACs are associated with increased *Akkermansia* sp. abundance; however, it is unknown whether microbiota changes are a direct action of PACs or an indirect result of their effects on host metabolism (151). B-type PACs are known to be less inhibitory to both bacteria and host metabolism (150). Work from our group demonstrates that pea seed coats rich in B-type PACs lead to a significant decrease in the Firmicutes population, increased fecal mucin content, and caused greater pathogen colonization in mice compared to a PAC-poor diet (152). B-type PACs may have led to improper mucus formation leading to a greater concentration of mucin excreted in feces. Phytonutrient supplementation is associated with increases in beneficial Clostridia species and can strengthen mucosal barrier function by increasing mucus production and thickness (153), protecting epithelial cells from invading pathogens and disease. Interestingly, a positive feedback loop may be established between mucolytic bacteria such as *Akkermansia* sp. that can degrade mucus O-linked glycans, thereby producing SCFAs that could stimulate goblet cells to secrete more mucus (14). Polyphenolic compounds may stimulate the microbiota directly or indirectly through modulation of mucus production, however further research is needed to establish direct links between diet and infection resistance (Figure 3).

## Vitamins and Minerals

Micronutrients are essential for proper metabolic and immune function. Nutrient and mineral deficiencies, typical in those that are critically-ill and in developing countries, can lead to metabolic changes, oxidative damage, immunological defects, weakness, and death (154). The effects of essential minerals, including iron, zinc, copper, selenium, silver, sulfur, calcium, phosphorus, and magnesium have been shown to affect resident microbial populations and health outcomes in both animal and human studies (155). Phagocytes have been shown to utilize the bactericidal actions of copper and zinc to enhance intracellular killing of pathogens (156). For instance, mice fed a zinc-deficient diet and challenged with Enteroaggregative *E. coli* (EAEC) had reduced leukocyte infiltration and increased virulence factors in luminal content, indicating an impaired immune response and increased infection severity (157). Regular supplementation of vitamin C (1–2 g/day) and zinc (<100 mg/day) reduced the duration of the common cold by 8–14 and 33%, respectively (158, 159). For vitamin C, prophylactic doses >0.2 g/day alleviated respiratory associated problems, particularly in physically strained and stressed individuals, however, its use as a therapy to treat the common cold remains controversial (160). In contrast, zinc supplementation studies support its use as a treatment option to reduce the duration and severity of the common cold (159). Vitamin D had the best overall protection against the common cold, however baseline vitamin D levels and dose must be considered since lower doses and deficient individuals experienced the most benefit (158). More mechanistic research is required to understand the impact of vitamins on immune responsiveness, especially with respect to the microbe-host gut axis in deficient and in excess conditions. Experiments in germ-free, conventionalized and infectious *C. rodentium* mice models confirm that the microbiota influences vitamin D metabolism by lowering fibroblast growth factor (FGF) 23 through increased activation of TNF-α in the colon (161). The fact that the presence of the microbial community or mono-colonization with *C. rodentium* increases serum vitamin D levels highlights their role on host homeostasis, especially since vitamin D levels control calcium homeostasis and bone formation (161). Research suggests that proper regulation of vitamins and minerals is key for establishing a proper immune response and intestinal barrier function. Similar to vitamin and mineral deficiencies, excessive supplementation can impair a host ability to resist enteric infections by altering intestinal integrity or enhancing pathogen fitness.

Recently, oral iron and vitamin B12 supplementation are suggested to impair microbiota dependent infection resistance. A systematic review and meta-analysis comprising 6831 adult participants concluded that oral ferrous sulfate (iron) supplementation is associated with a significant increase in gastrointestinal side-effects compared to placebo and intravenous iron delivery (162). This reveals that the effects of iron supplementation are possibly initiated through the microbe-gut axis with unknown consequences and should be used cautiously. For instance, excessive luminal iron affects intestinal integrity through oxygen radical production, encourages pathogen virulence, and alters microbial populations leading to pathogen overgrowth (163, 164). In a dose dependent manner, iron increased epithelial invasion and translocation of *S. typhimurium* in Caco-2 cells *in vitro* and reduced the survival of the nematode *Caenorhabditis elegans* infected with *S. typhimuriumi* (163, 165). Regulation of luminal iron is extremely important for maintaining intestinal integrity and controlling pathogen expansion (166). Furthermore, lipocalin-2 is a protein produced by neutrophils and epithelial cells during inflammation that directly limits bacterial iron uptake, reducing pathogen overgrowth and severity (167). Unlike iron, vitamin B12 is directly regulated in the gut by intrinsic factors for absorption and in excess, it can escape host absorption and affect microbial competition. The gut commensal bacteria *Bacteroides thetaiotaomicron* may compete against enterohemorrhagic *E. coli* (EHEC) to sequester dietary vitamin B12 (168). *In vitro* competition assays show that *B. thetaiotaomicron* reduced EHEC shiga toxins but when co-cultured with a mutant *B. thetaiotaomicron* lacking a vitamin B12 transporter, EHEC had normal shiga toxin production (168). Microbial vitamin B12 transporters have different affinities toward vitamin B12 allowing them to compete with host cells and other microbes to take up exogenous vitamin B12 (169, 170). More research is needed into micronutrient supplementation on host-microbe interactions toward pathogens, especially in the context of over-supplementation, which may be detrimental depending on the micronutrient balance and host intestinal homeostasis. Limiting the expansion of enteric pathogens can be accomplished by reducing their access to vitamin or minerals either through diet or stimulation of gut commensals to compete with pathogen for vital nutrients.

## Conclusion

Pathogen resistance and tolerance requires tight host regulation of dietary components and subsequent microbial actions that together influence each other and host immunity. Undigested and unabsorbed dietary components are able to influence microbial populations and their fermentation by-products can indirectly contribute to infection resistance by modulating host intestinal integrity. Dietary intervention studies are difficult to control and compare due to seasonal variations in diets sources. We suggest that dietary intervention studies should include diet backgrounds designed with macro- and micro- nutrients that stress and protect the gastrointestinal environment, as to give a proper assessment of that dietary component on host. In general, a balanced diet of SFA, MUFA, MACs, protein, phytochemicals, vitamins, and minerals with limited sources of n-6 PUFAs, simple carbohydrates, BAPs, and iron may help restore intestinal homeostasis in compromised individuals. Dietary individuality makes it difficult to make general diet recommendations as each individual may have genetic, microbiota, and unforeseen environmental factors that influence diet digestibility and utilization. Together, these factors ultimately provide the context to which dietary components may influence intestinal integrity and homeostasis.

## Author Contributions

The concept of this review was developed by BW and AF. This review was written by AF and JF, and was edited by BW, AF, and JF.

### Conflict of Interest Statement

The authors declare that the research was conducted in the absence of any commercial or financial relationships that could be construed as a potential conflict of interest.
